# Electrospun PCL Filtration Membranes Enhanced with an Electrosprayed Lignin Coating to Control Wettability and Anti-Bacterial Properties

**DOI:** 10.3390/polym16050674

**Published:** 2024-03-01

**Authors:** Sara Bergamasco, Noemi Fiaschini, Luis Alexander Hein, Marco Brecciaroli, Roberta Vitali, Manuela Romagnoli, Antonio Rinaldi

**Affiliations:** 1 Department for Innovation in Biological, Agro-Food and Forest Systems (DIBAF), University of Tuscia, Via San Camillo de Lellis snc, 01100 Viterbo, Italy; sara.bergamasco@unitus.it; 2 NANOFABER S.r.l., Via Anguillarese 301, 00123 Rome, Italy; noemi.fiaschini@nanofaber.com (N.F.); luis.hein@nanofaber.com (L.A.H.); 3 Simitecno srl, 00173 Rome, Italy; marco.brecciaroli@simitecno.it; 4 SSPT-TECS-TEB Laboratory, ENEA—Italian National Agency for New Technologies, Energy and Sustainable Economic Development, Via Anguillarese 301, 00123 Rome, Italy; roberta.vitali@enea.it; 5 SSPT-PROMAS-MATPRO Laboratory, ENEA—Italian National Agency for New Technologies, Energy and Sustainable Economic Development, Via Anguillarese 301, 00123 Rome, Italy

**Keywords:** electrospinning, electrospraying, lignin, oak, eucalyptus, PCL, FTIR, SEM, morphology, wettability, antibacterial test

## Abstract

This study reports on the two-step manufacturing process of a filtration media obtained by first electrospinning a layer of polycaprolactone (PCL) non-woven fibers onto a paper filter backing and subsequently coating it by electrospraying with a second layer made of pure acidolysis lignin. The manufacturing of pure lignin coatings by solution electrospraying represents a novel development that requires fine control of the underlying electrodynamic processing. The effect of increasing deposition time on the lignin coating was investigated for electrospray time from 2.5 min to 120 min. Microstructural and physical characterization included SEM, surface roughness analysis, porosity tests, permeability tests by a Gurley densometer, ATR-FTIR analysis, and contact angle measurements vs. both water and oil. The results indicate that, from a functional viewpoint, such a natural coating endowed the membrane with an amphiphilic behavior that enabled modulating the nature of the bare PCL non-woven substrate. Accordingly, the intrinsic hydrophobic behavior of bare PCL electrospun fibers could be reduced, with a marked decrease already for a thin coating of less than 50 nm. Instead, the wettability of PCL vs. apolar liquids was altered in a less predictable manner, i.e., producing an initial increase of the oil contact angles (OCA) for thin lignin coating, followed by a steady decrease in OCA for higher densities of deposited lignin. To highlight the effect of the lignin type on the results, two grades of oak (AL-OA) of the *Quercus cerris* L. species and eucalyptus (AL-EU) of the *Eucalyptus camaldulensis* Dehnh species were compared throughout the investigation. All grades of lignin yielded coatings with measurable antibacterial properties, which were investigated against *Staphylococcus aureus* and *Escherichia coli*, yielding superior results for AL-EU. Remarkably, the lignin coatings did not change overall porosity but smoothed the surface roughness and allowed modulating air permeability, which is relevant for filtration applications. The findings are relevant for applications of this abundant biopolymer not only for filtration but also in biotechnology, health, packaging, and circular economy applications in general, where the reuse of such natural byproducts also brings a fundamental demanufacturing advantage.

## 1. Introduction

Water filtration using separation membranes is one of the methods employed for wastewater treatment, where such membranes act as a physical barrier, allowing the passage of water molecules while sieving molecules, microorganisms, and dissolved substances larger than a given cutoff [1]. Polymeric membranes produced by electrospinning have shown significant potential for water filtration applications, garnering substantial interest over time due to their ability to create highly customizable materials with controlled porosity [2]. In fact, electrospinning is an advanced manufacturing method of great interest in the filtration industry as it enables the production of either self-standing fibrous non-woven membranes or just coatings characterized by high porosity, open-pore microstructure, and a large surface area [3]. The interconnected percolating pores within a fibrous electrospun layer are capable of enhancing filtration capacity [4] and can be easily tailored in pore size and overall void fraction to modulate the filtration performance to achieve selective removal of target contaminants, such as particulates [5], heavy metals [6], oils and organic compounds [7], and microbial agents like bacteria and viruses [8]. Electrospinning is recognized as a very versatile technique for processing various polymers to create nanofiber-based membranes and coatings, but it can also be used in electrospray mode to produce emulsions or coatings made of particles made of micronized polymer [9]. 

Several technological polymers are deployed in the electrospinning of filtering media. For example, polyacrylonitrile (PAN) is used for its excellent chemical, mechanical, and thermal properties [10]. This polymer is often used in synergy with other materials; e.g., Perekh et al. [11] produced electrospun PAN membranes incorporating silver nanoparticles to provide clean and drinkable water for underdeveloped countries. Polyvinylidene fluoride (PVDF) is another example of an interesting material with characteristics attractive for direct osmosis applications in seawater desalination [12]. Polysulfone (PSU) is also commonly used in this field, both alone and in combination with other materials [13]. For instance, PSU membranes coated with a layer of magnetite nanoparticles enabled the removal of model viruses (i.e., bacteriophage MS2) [14], whereas adding a nanocoating of silver nanoparticles endowed PSU electrospun membranes with antibacterial properties [15]. Yet, in the mainstream transition to a circular economy, the development of a new class of more sustainable electrospinning materials is becoming a key research objective. 

In recent years, membranes made of poly(ε-caprolactone) (PCL) have been investigated as a relatively environmentally friendly option for water treatment applications [16]. Despite being a synthetic polymer, PCL possesses desirable properties such as biodegradability and biocompatibility [17]. These attributes are crucial for water treatment, as biodegradability allows for the degradation of PCL into products such as capronic acid, succinic acid, valeric acid, and butyric acid [18]. by the action of microorganisms, making them environmentally friendly and suitable for disposal without causing harm to the environment [19]. From the viewpoint of circular economy, this makes PCL a potential candidate to implement the demanufacturing of exhaust membranes and help address the end-of-life problems of current products. Biocompatibility is another important pre-requisite for safe exploitation, ensuring that the material is non-toxic to both humans and the environment [20]. Furthermore, PCL is easily workable, offers excellent flexibility when combined with other polymers to modify its properties, and exhibits good solubility in a wide range of organic solvents commonly used in electrospinning processes [21].

In the field of water filtration, reportedly, PCL was deployed for the fabrication of composite electrospun membranes with high performance when used in combination with cellulose nanofibrils (CNF) [22]. In that case, the CNF enhanced the mechanical properties of the composite fibers and helped improve the filtration quality in terms of turbidity, conductivity, and the removal of metallic contaminants. This approach to membrane design is interesting as it yields a system that pairs a filtration performance tailorable by a functional additive (i.e., CNF in this example) to the intrinsic demanufacturing proneness of the PCL matrix. In another example, nanofibrous membranes made of chitosan and PCL delivered a composite architecture inheriting the antimicrobial properties of chitosan in combination with the advantageous characteristics of PCL [23]. In another example, hybrid nanocomposite membranes of PCL/TiO_2_ were investigated for the treatment of wastewater from the dairy industry, showing that such membranes could combine the hydrophobic properties and chlorine resistance of PCL with the antibacterial and anti-fouling properties associated with the hydrophilicity of titanium dioxide [24]. Finally, our group also investigated several composite electrospun systems based on PCL, such as the PCL/Ag endowed with a Ag nanocoating of PCL fibers via magnetron sputtering [25]. In conclusion, the design and manufacturing of advanced composite membranes obtained via electrospinning do represent a timely and rewarding exploration area. 

In this paper, we investigate the combination of PCL with lignin, a new biomaterial that is attractive and is currently receiving significant attention. Lignin is, in fact, the second most abundant natural polymer in nature [26] and represents a renewable and sustainable resource that can be harvested from several feedstocks of the wood industry. Lignin finds diverse applications across various industrial fields, including the coating industry for its UV protection capabilities [27], the synthesis of therapeutic hydrogels [28] for their antibacterial properties and biocompatibility, and as a precursor for high-value-added product manufacturing [29]. Additionally, the abundance of functional groups makes lignin suitable for the production of flocculants, adsorbents, and dispersants [30]. All these properties make lignins a worthy candidate to explore for water treatment applications.

Hereafter, we present a new 3-layered composite membrane consisting of a backing of filtering media, covered with a thick PCL layer inspired from our prior study [25], and here functionalized by an innovative biocoating made of lignin via an electrospinning process driven in electrospray mode. We recall that electrospinning and electrospraying coexist on the same technological platform and just represent two regimes of the same electrohydrodynamic technique, leading to two different outcomes depending on the polymer solution being processed. Electrospinning and electrospraying represent cutting-edge methodologies for producing nanofibers and particles with controlled dimensions down to the nanoscale, using electric fields on the order of kilovolts per centimeter. These processes offer broad versatility in structure design, enabling manipulation of key parameters [31] to achieve the desired morphology. These parameters can be classified into factors related to the solution [32], operational parameters like applied electric field [33] and working distance [34,35], and environmental factors such as temperature and relative humidity. In this article, we do not aim to delve into the theoretical chemical-physics aspects underlying these phenomena, as they are not the focus of the presented work. However, we aim to provide some basic information on the main practical differences between the employed techniques. As widely recognized, the nanofiber formation process involves a series of complex phenomena [36], ranging from the initial formation of a straight jet from the solution, with the development of the Taylor cone, to the solidification of the polymeric material during its flight towards the collection substrate. The jet’s expulsion is influenced by various forces, including surface tension, viscoelastic force, repulsive forces, air drag force, and gravity [37]. Furthermore, the driving force responsible for fiber generation is provided by the electrical potential difference between the injector and the collector. 

In synergy with these aspects, the intrinsic properties of the polymeric solution play a crucial role. For example, each solution has a critical concentration value above which fiber formation is initiated, while lower concentrations allow for the production of monodisperse particles [38]. The polymer molecular weight, closely related to the solution concentration value, determines the formation of fibers or dispersed particles. Based on the choice of the polymer’s molecular weight and the concentration of the solution, another important parameter arises, namely the final viscosity of the electrospun solution. In cases of low molecular weights and low-concentration solutions, as well as in the case reported here, a homogeneous spray of the dispersed polymer is achieved, while increasing viscosity pushes the process towards fiber production, as illustrated by McKee et al. [39], who describe the correlation between solution rheology and the formation of electrospun fibers. Finally, electrical conductivity [40] can interact with gravity and electrical forces, contributing to the formation of conical jets. Careful management of these parameters and solution properties is essential for achieving the desired results. 

As there are many lignin grades, two different types of lignin are investigated and benchmarked here, namely acidolysis lignin (AL) from oak wood (AL-OA) and from eucalyptus wood (AL-EU) [41]. Morphological, physical, and antibacterial studies were conducted on prototypical membranes, varying not only the type of lignin but also altering the electrospraying deposition time, with five different time points selected to monitor the evolution of the composite membrane properties and microstructure vs. deposition time. The main goal is to show that pure lignin can be processed by electrospraying to obtain a functional coating with thickness from the nanometer scale to the micrometer scale, but the scope of our research also covers aspects related to the possibility of varying the amount of deposited lignin (i.e., thickness or areal density of the coating) to modulate the functional properties. The latter aspect is relevant to adjusting the final filtration product to the specific requirements of a given engineering application.

## 2. Materials and Methods

### 2.1. Materials

PCL Capa 6800D from Perstorp (UK Limited, Warrington, UK), Acidolysis lignin from eucalypt (*E. camaldulensis*) and oak (*Q. cerris*) species, N,N-Dimethylformamide (DMF) from Supelco, Merck KGaA (Darmstadt, Germany), Chloroform (CHCl_3_) stabilized with 2-methyl-2-butene, provided from VWR chemicals (Radnor, PA, USA), Qualitative filter paper 600 from VWR International (Radnor, PA, USA), Ethanol absolute from Merck KGaA (Darmstadt, Germany), deionized water, 1 Primary Wound Dressing© (1PWD) (CE 0344)—Phytoceuticals, Zurich, Switzerland). *Staphylococcus aureus* (ATCC 29213) and *Escherichia coli* (ATCC 25922) were used, respectively, as representatives for Gram-positive and Gram-negative pathogens.

### 2.2. Preparation of Solutions and Fabrication of Membranes through Two Steps Electrospinning

*Step 1:* Supported PCL membranes were electrospun on a selected paper filter media (600, VWR International, Radnor, PA, USA) to produce a microfiltration membrane using industrial electrospinning equipment (Fluidnatek^®^ LE100, Bioinicia, Paterna, Spain) at NANOFABER S.r.l., equipped with a large stationary planar collector and an environmental control unit, enabling to keep temperature at 25 °C and relative humidity (RH) at 40% throughout manufacturing. The electrospinning of PCL was performed according to the recipe of a commercially available product, the PCL-NBARE ™ series (NANOFABER S.r.l., Rome, Italy), for the solution electrospinning of PCL granules in a mixture solvent of DMF and chloroform [25]. The filtration paper covered with bare PCL represented the reference system throughout the study, referred to as “PCL” or “uncoated PCL” hereafter.

*Step 2:* For electrospraying the coating of lignin, two 5% (w,w) solutions of oak (AL-OA) and eucalypt lignin (AL-EU) in DMF were prepared. These two solutions were prepared using a thermostated bath at 60 °C and were magnetically stirred for two hours. The lignin solutions were subsequently allowed to stabilize at room temperature for two days before being processed through electrospinning. The fabrication of the final composite membranes was performed according to protocols by NANOFABER S.r.l., by adding to Step 1 a second processing step of electrospray of a surface coating of AL-EU or AL-OA onto the PCL layer. The two steps were performed sequentially in situ using said industrial electrospinning equipment. Several coated membranes of 19 × 24 cm^2^ in size were prepared for different sets of parameters, as listed in Table 1.

### 2.3. Morphology, Mechanical Testing and Roughness of the Membranes 

The morphological study of the membranes was conducted using a field emission gun scanning electron microscope (SEM) (Leo 1530, ZEISS, Jena, Germany) working at a low extraction voltage of 3.00 kV to avoid charging. Samples were analyzed without applying a conductive Au nanocoating. To measure the thickness of the membranes (*Th*), cross-sectional sections were placed between two silicon wafers. Three measurement points were taken for each cross-sectional section. In these measurements, a Zeiss AXIO Zoom.V16 microscope (ZEISS, Jena, Germany) was used, and images were processed using Zen Blue Version 3.4.91 software. In addition to image-based analysis, an experimental assessment of membrane porosity was conducted using the displacement volume technique using absolute ethanol. 

The mechanical properties of the filter paper and of the PCL layers alone were evaluated by tensile tests, similarly to prior work [15]. A macro-tensile loading frame (MICROTEST 200 N, DEBEN, Suffolk, UK) equipped with a 200 N load cell was used to test rectangular strips cut out of samples. The Young modulus (E) and the ultimate tensile strength (UTS) of each material were measured from their stress vs. strain curve, respectively, as the slope in the linear response region and as the maximum tensile stress reached before either the rupture or the onset of the softening phase.

The roughness value Ra (Arithmetic Average Roughness) for each sample was evaluated using NPS confocal scanning (NP1 probe, HIROX, Tokyo, Japan), a methodology compliant with ISO 21920 [42].

### 2.4. Porosity and Permeability Determination

The porosity of the membranes was assessed using the liquid displacement method [43,44]. Ethanol (Merck, Darmstadt, Germany) was selected as a penetrating liquid due to its ability to infiltrate through the porous membrane, coupled with a lower density (ρ_EtOH_ ≅ 0.790 g/mL). The percent porosity (ε_%_) was computed from Equation (1):

ε_%_ = (m3 − m4 − m1)/(m2 − m4) × 100 (1)

by determining the weights m1, m2, m3, and m4 on a scale (ORMA, BCA120, Milan, Italy) at a temperature of 20 °C. For a given membrane specimen, m1 represents the “as-dry” weight, m2 represents the weight of a “control” graduated bottle filled with ethanol up to a specified “control volume”, and m3 is the weight of the same bottle after the membrane specimen is inserted and immersed in the ethanol, with the displaced excess volume of ETOH carefully removed using a pipette to restore the control volume. Finally, m4 reflects the weight of the bottle containing the residual ethanol after the wet membrane is extracted.

The assessment of air permeability for the electrospun membranes was conducted using a Gurley densometer (model 4320, Troy, NY, USA). In this test, the time (*t*) in seconds required for a volume (*V*) of air equal to 100 cm^3^ to pass through a surface (*A*) with a fixed area of 1.58 cm^2^ was measured. Equation (2) renders the permeability value (*P*) for each sample in “cm/s” [45].

(2) $$P=\frac{V}{A\times t}$$

### 2.5. Wettability Tests of Membranes

The wettability tests of the membranes were conducted by measuring the contact angle using a Leica Wild M3Z (part of Hexagon, Stockholm, Sweden) stereomicroscope. Images were captured using a Moticam 2000. For the contact angle analysis, two representative liquids were used: deionized water (WCA) to represent the hydrophilic part and “1PWD” (1 Primary Wound Dressing©) to represent the lipophilic part (OCA). The measurement of the contact angle between the membrane and a droplet of the liquid was performed 10 s after depositing 10 μL of the liquid onto the membrane surface.

### 2.6. ATR-FTIR Analysis

For the ATR-FTIR (Attenuated Total Reflection Fourier-Transform Infrared) analysis of the samples, a Nicolet iS50 spectrometer connected to the iD70 ATR accessory (Thermo Fisher Scientific, Waltham, MA, USA) was used. The crystal used for this ATR analysis was a monolithic diamond crystal. The spectra were recorded in absorbance mode with a resolution of 4 cm^−1^. Each sample was scanned 16 times in the wavelength range from 4000 to 400 cm^−1^.

### 2.7. Antimicrobial Test

Circular PCL disks with a diameter of 15 mm, created through the die-cutting process, were placed in 24-multiwell plates for *Staphylococcus aureus* (*S. aureus)* and *Escherichia coli* (*E. coli)* biological tests. *S. aureus* and *E. coli* stock cultures kept at −80 °C in 10% (*w*/*v*) glycerol (Merk Life Science S.r.l., Milan, Italy) were inoculated, respectively, into 3 mL of Nutrient broth (Biolife Italiana, Milan, Italy) and of Luria Bertani (LB) broth (Merck Life Science S.r.l., Milan, Italy). Both bacterial cultures were incubated at 37 °C O/N with constant shaking at 180 rpm before their use in experiments. The day of the test, the *S. aureus* bacterial concentration was diluted and grown until OD600 was 0.02, about 1.1 × 10^6^ colony forming units/mL (CFU/mL); instead, the *E. coli* bacterial concentration was diluted and grown until OD600 was 0.028, about 5.0 × 10^6^ colony forming units/mL (CFU/mL). Subsequently, the bacteria were introduced into a 24-well multiwell plate, where they were exposed to either uncoated PCL samples (blank disks) or Lignin-coated samples. The incubation took place at 37 °C under continuous agitation at 50 rpm. Two types of experiments were performed.

In the first experiment, a control with *S. aureus* alone was also inserted, and the samples were incubated with the microbial population. The survival rate was determined at 1.5 h and 3 h using the count plate method and the calculation of CFUs to determine the cytocitic action of the samples.

In the second experiment, at times t = 1 ½, 3, and 4.5 h, 10 μL of each sample were diluted in phosphate-buffered saline (PBS) (Euroclone, Milan, Italy) (serial dilution from 10^−1^ to 10^−8^) and 100 μL of each dilution were distributed on Nutrient agar dishes (15 g L^−1^ agar) (Panreac Química SLU, Barcelona, Spain) for *S. aureus* strain and on Luria Bertani agar (15 g L^−1^ agar) dishes for *E. coli* strain. All dishes were incubated for 18 h at 37 °C. Following the incubation period, colonies that had reached a size discernible bare-eye were enumerated, and the concentration corresponding to the counted colonies was determined. 

The antibacterial experiments were replicated twice for consistency and reliability of the results. The acquired data were expressed as the mean ± SEM. Prism 7 software was utilized to perform Student’s *t*-test. Significant differences were highlighted for two significance levels as * *p* < 0.05 or ** *p* < 0.01.

## 3. Results and Discussion

**Figure 1:**
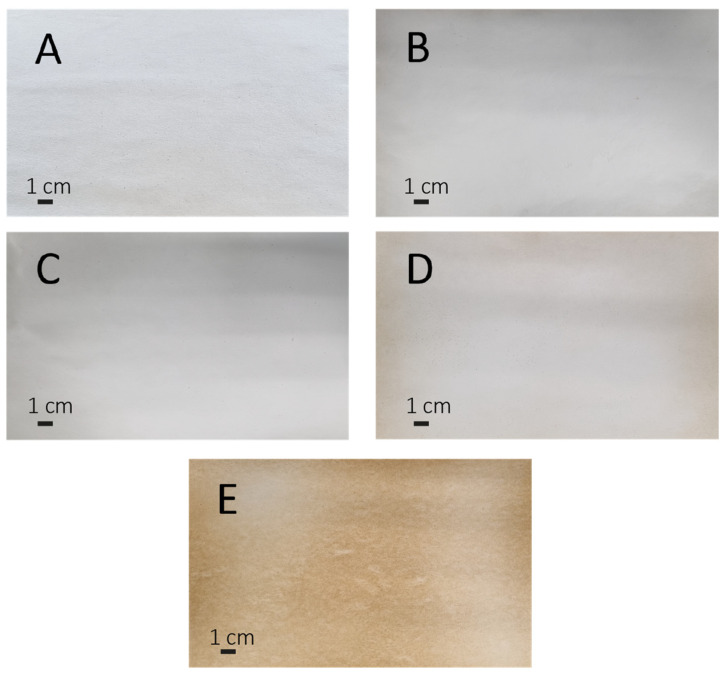
Representative pictures of PCL membranes coated with eucalypt lignin at different times showing color changes as the deposition time increases: (**A**) bare PCL; (**B**) AL-2.5; (**C**) AL-5; (**D**) AL-10; and (**E**) AL-60.

Self-standing membranes consisting of filter paper and an electrospun PCL layer were used as the control and as the basis for subsequent treatments obtained by performing a second coating step with lignin. At the end of all manufacturing processes, 11 membranes were produced (refer to examples in Figure 1), and coatings were produced at five different deposition times for each of the two lignin grades, as summarized in Table 2.

### 3.1. Microstructure, Mechanical Properties and Roughness of Membranes 

**Figure 2:**
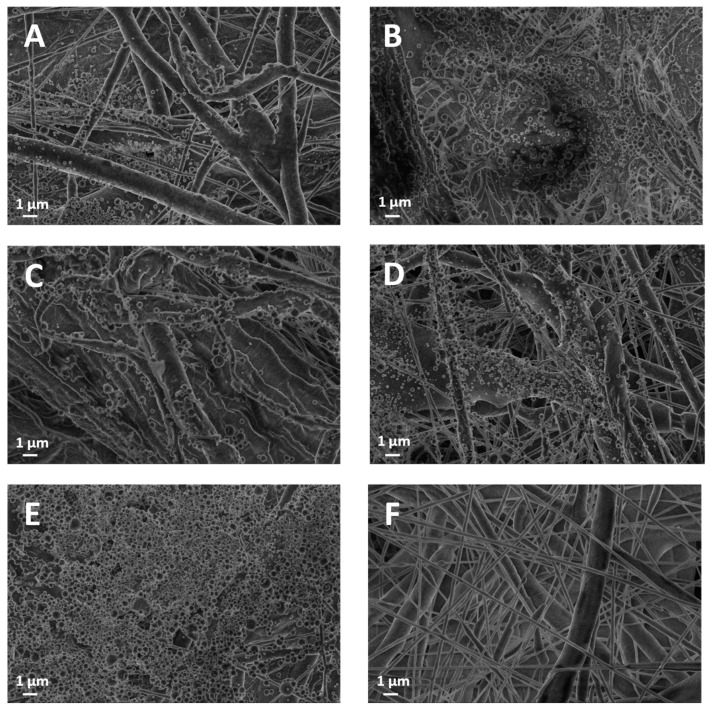
Scanning Electron Microscope (SEM) results: (**A**) AL-EU-2.5; (**B**) AL-EU-5; (**C**) AL-EU-10; (**D**) AL-EU-60; (**E**) AL-EU-120; and (**F**) PCL.

The investigation of our multilayer samples started with the microstructural analysis of surface texture (of the electrospun material) and thickness. The SEM images in Figure 2 portray the architecture of the PCL non-woven fibers along with the distribution of lignin sprayed on top of them, which appears more pronounced with deposition time. Up to 60 min, the mesh structure of PCL fibers is still clearly visible, but for a deposition time of 120 min, the lignin coating becomes conformal and completely shields the underlying fibrous structure. Interestingly, although deposition time is proportional to the spatial density of lignin particles, the size distribution of said particles is seemingly invariant and estimated to be approximately 320 ± 130 nm (mean ± SD) for both lignin grades and for any deposition time. In contrast, the diameter distribution of the randomly oriented PCL fibers was about 240 ± 150 nm (Figure 2F), indicating that PCL fibers and lignin particles have a similar characteristic length scale. In the supplementary materials, SEM images depict the thickness variations of PCL membranes coated with electrosprayed lignin.

**Figure 3:**
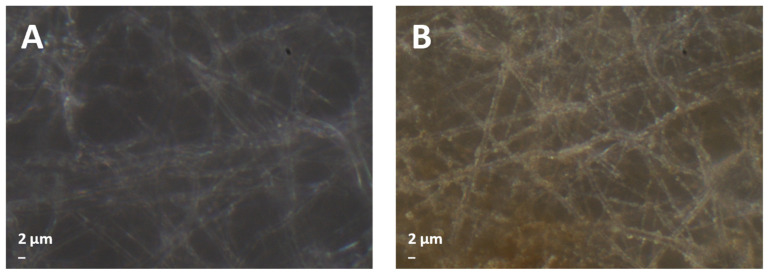
Illustrative optical microscope images: (**A**) PCL coated for 10 min with AL-OA; (**B**) PCL coated for 120 min with AL-OA.

Figure 3 shows the optical microscope images of PCL membranes coated for 10 min with AL-OA (A) and PCL coated for 120 min with AL-OA (B).

Regarding thickness measurements (*Th*), the average and SD for each sample are summarized in Table 3 (data net of the nominal filter paper support thickness equal to 110 µm for all cases), but do not offer an immediate interpretation for the observed trends. PCL/AL samples for coating times of 2.5 and 5 min, both with oak and eucalyptus lignin, exhibit thickness values similar to each other.

**Figure 4:**
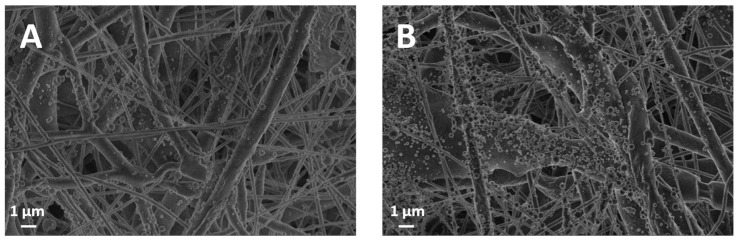
SEM micrographs of AL-OA-60 (**A**) and AL-EU-60 (**B**), the latter already shown in Figure 2D, indicate that lignin nanoparticles tend to coalescence more markedly in the case of eucalyptus lignin than for oak lignin.

However, everything else being equal, for a 10-min deposition time (AL-10), the thickness of the PCL membrane with eucalyptus lignin (49.75 μm) is lower compared to oak lignin (65.28 μm). This difference can be attributed to the intrinsic nature of the extracted lignins, with eucalyptus lignin being richer in lipophilic extractives. Before the acidolysis chemical treatment for lignin recovery, the woody biomasses were in fact pretreated with an acetone/water mixture to remove wood extractives, as reported by Bergamasco et al. [41]. While this chemical treatment enabled the removal of all wood extractives from both species, it is not selective with respect to lipophilic components. It is known that for the recovery of these hydrophobic components, the use of solvents such as dichloromethane [46] or n-hexane [47] is preferable. Then, we can assume that hydrophobic components are still present in a minimal percentage and that AL-EU contains more of them because lipophilic extractives were detected in the FTIR analysis previously reported [41]. These lipophilic components, as supported by the SEM image at the 60-min lignin deposition time (Figure 4B), seem to contribute to the formation of clusters of AL-EU nanoparticles, unlike the PCL-coated AL-OA sample (Figure 4A), where the nanoparticles are well separated. These oily clusters could interact with the PCL membrane, which demonstrated a hydrophobic nature following the analysis of the contact angle reported later herein, somehow permeating and intercalating between the PCL fibers and thus reducing the overall thickness of the obtained membrane.

As deposition time increases, this asymmetry between OA and EU disappears, and both AL-120 samples display a mild decrease in thickness as a direct consequence of the extended AL coating time.

In Table 4, the estimated values of the AL coating thickness achieved for different amounts of lignin deposited per cm^2^ of membrane are presented. To back-calculate the areal density (in grams of lignin per cm^2^), we considered the concentration of the initial solution, the volume of solution required for the coating, and the nominal membrane surface area (19 × 24 cm^2^). Since the volume of lignin deposited from electrospinning is known and the reported lignin density is about 1.4 g/cm^3^ [48], the thickness values in Table 4 are obtained. While these are approximate numbers, the AL-coating for shorted deposition times (2.5 and 5 min) is indeed nanocoating with a thickness around or below 50 nm and an area density of 30–60 g/m^2^.

From a structural viewpoint, the mechanical integrity of the entire filter relies entirely on the filter paper. In fact, neglecting the thin lignin layer, the filter paper is thicker and has higher performance (*E* = 18.8 MPa and *UTS* = 26.4 MPa) than PCL (*E* = 0.15 MPa and *UTS* = 1.9 MPa), such that the contribution of PCL to the load-bearing capability of the whole filter system under uniaxial tensile stress is estimated to be less than 1%, as computed from the ratio $E_{PCL}\cdot {Th}_{PCL}/(E_{PCL}\cdot {Th}_{PCL}+E_{PAPER}\cdot {Th}_{PAPER})$. 

**Figure 5:**
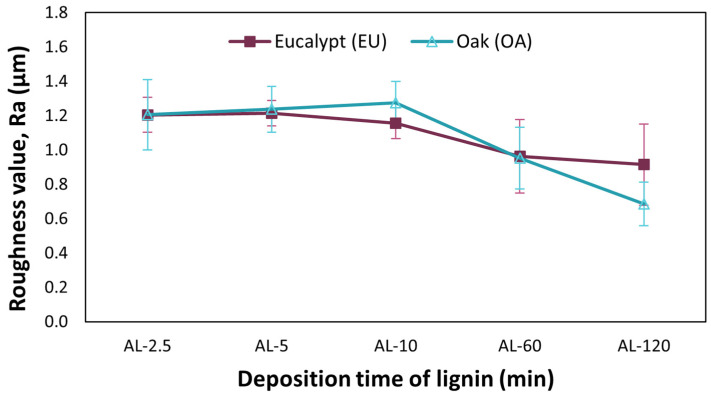
Trends in roughness Ra (Arithmetic Average Roughness) as a function of coating time with different lignins.

Figure 5 illustrates the trend of the average roughness (Ra) as a function of the increasing coating time of lignin. A decrease in the average roughness (Ra) is generally observed with an increase in the coating time. In particular, it is observed that for coating times up to 5 min, the average value of Ra tends to remain almost constant, close to 1.2 µm for both lignin grades used. After 10 min of coating, in the case of EU, the roughness decreases to 1.16 µm, while in the case of OA, a slight increase to 1.27 µm is measured. For long lignin coating times, the roughness value decreases and converges to 0.95 µm for both EU and OA after 60 min. After 120 min, in the case of EU, the roughness decreases slightly to 0.92 µm, while in the case of OA, the decrease in roughness is greater, dropping to 0.687 µm.

### 3.2. Porosity and Air Permeability Analysis 

**Figure 6:**
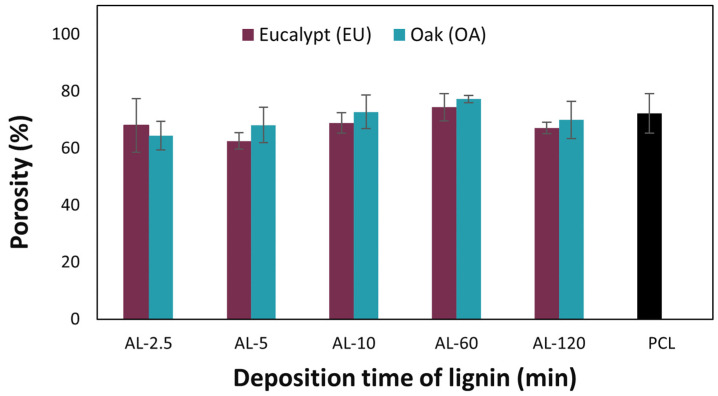
Trends in porosity of the layered membrane for each lignin grade and for different deposition times.

The analysis of the overall porosity of the coated membranes was conducted using the volume displacement method, and the average values obtained from three measurements for each membrane are presented in Table 5. As seen in Figure 6, the porosity of the PCL-coated membrane without lignin nanocoating was found to be 72.22%, a value consistent with what is reported in the literature, where such membranes typically exhibit porosity ranging between 60% and 90% [49]. The membranes containing lignin on the surface did not show significant deviations in porosity, which is to be expected because the lignin coating is much smaller than the PCL layer, as reported in Table 5. 

While the influence of the spray-coating step on porosity is negligible for all five deposition times under consideration, data indicates that for increasing deposition times (ref. AL-120 min in Figure 2 and AL-60 in Figure 4), the lignin nanoparticles tend to overlap and accumulate, filling in some of the pores underneath in the PCL layer. The relationship between the decrease in porosity and the formation of a conformal surface coating, confirmed by SEM for both AL-EU and AL-OA at 120 min, provides a rationale for the decrease in porosity for a longer deposition time of lignin vs. the reference PCL membrane. This is in agreement with the literature, and, for example, Ahmadpour et al. [50] reported a surface modification of a membrane composed of polyether imide (PEI) by a fine spray of material, leading to a reduction in the porosity of the filtration membrane. 

**Figure 7:**
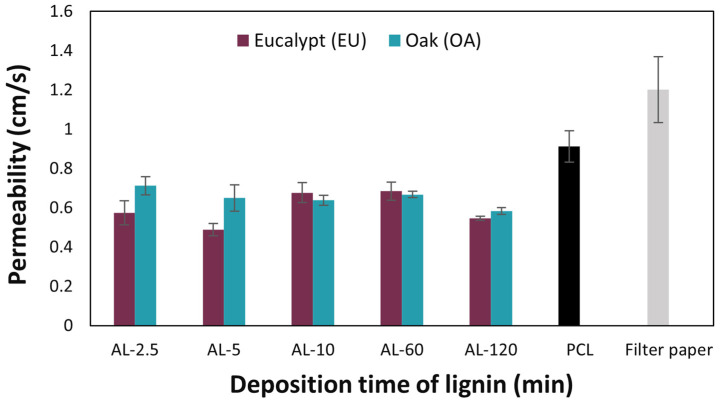
Comparison of permeability results (cm/s).

In addition to porosity, permeability tests offer technological data that is relevant to the understanding of the linkage between microstructure and performance. The study conducted using the Gurley densometer revealed a significant reduction in the air permeability of the membranes following the coating with lignin spray compared to sole PCL (Table 6 and Figure 7). For short coating times, the membranes exhibited permeability values significantly lower than average, especially when eucalyptus lignin was used, with permeability values of 0.57 at AL-2.5 and 0.49 cm/s at AL-5. This phenomenon could not be attributed to the intrinsic structure of the PCL membranes themselves before the coating with eucalyptus lignin. These membranes appear to already possess a structure that offers greater resistance to permeation compared to the control. However, sweeping for increasing deposition time, from AL-10 to AL-120, the permeation data show remarkable similarity between the two lignins under study. Interestingly, in the case of AL-10 and AL-60 times, these membranes exhibit similar behavior in terms of permeability despite the significant difference in coating times. The SEM (Figure 2 and Figure 4) indicated that in general, the sprayed lignin particles tend to accumulate mainly on the surface of the PCL fibers, leaving the pores that make up the mesh structure of the membranes apparently unobstructed. However, as already mentioned, this is no longer the case for AL-120 membranes, where the situation is significantly different, and SEM shows lignin particles completely covering the membrane surface and masking the fibrous PCL microstructure underneath. The formation of such a homogeneous and dense coating at the microscopic level is reflected by the permeability values for eucalyptus (0.55 cm/s, AL-EU-120) and oak (0.58 cm/s, AL-OA-120) lignin coatings, which are significantly lower than reference membrane (0.91 cm/s).

### 3.3. Contact Angle 

To assess the hydrophilic/hydrophobic nature of PCL membranes uncoated and coated with lignin at different deposition times, wettability tests were performed both for polar (i.e., water) and apolar (i.e., a 1PWD oil—1 Primary Wound Dressing© oil). The results in Table 7 demonstrate that the lignin coating leads to a significant modification of the typically hydrophobic character of PCL membranes [25]. 

**Figure 8:**
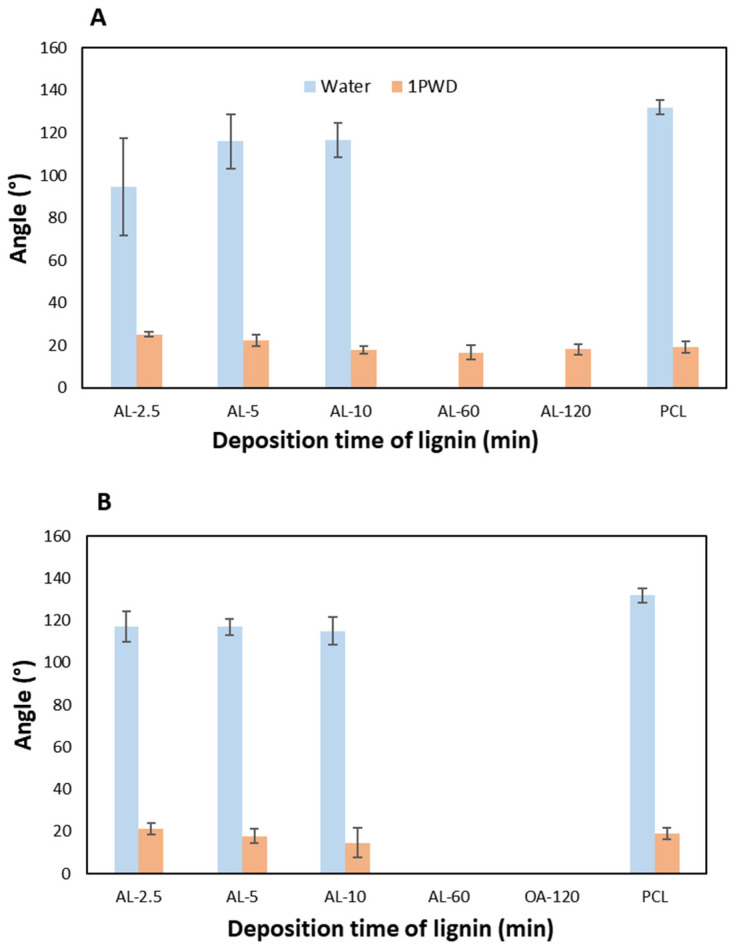
Contact angle histograms of AL-EU (**A**) and AL-OA (**B**) measured using water and 1PWD (1 Primary Wound Dressing©).

Water wettability tests on both AL-EU (Figure 8A) and AL-OA (Figure 8B) coatings decrease the water contact angle (WCA) compared to bare PCL membranes for electrospraying deposition times of 2.5, 5, and 10 min. For deposition times of 60 and 120 min, the effect is even more drastic and could not be measured with our method (i.e., 0° values in Table 7) because the water droplet completely disappeared, being quickly absorbed into the membrane within the set time (10 s) of the test and revealing a transition to full hydrophilicity of the surface. This behavior can be attributed to the abundance of polar hydroxyl and carboxylic groups present in the structure of the lignin nanoparticles [51,52,53]. This result is of particular technological interest as it is possible to modulate the hydrophilicity of PCL membranes by a small lignin amount, i.e., short deposition times. In turn, the possibility of delivering a substantial increase in hydrophilicity for rather short coating times is crucial for the deployment of a new “green” coating technology in high-throughput manufacturing, such as in the filtration industry and textile applications. 

The scenario from oil wettability tests is quite different, since the PCL layer is very affine to oil and exhibits contact angle values much lower than water (19° vs. 131°). By dispensing droplets of 1PWD (1 Primary Wound Dressing©), a liquid oil markedly hydrophobic, the oil contact angle measurements (OCA) did not significantly differentiate from the PCL control, with a few notable exceptions. In the case of AL-EU-2.5 and AL-OA-2.5 coatings, an increase in OCA was observed. More importantly, for oak lignin, in the cases of AL-OA-60 and AL-OA-120, it was not possible to measure the contact angle due to the complete absorption of the 1PWD droplet within the set time. Conversely, the corresponding measurements for eucalyptus at 60 and 120 min appeared to be in trend without dropping to 0°. The reason for such a difference is unclear, although a possible explanation can be found in the different chemical structure of lignin. As highlighted by Py-GCMS analyses conducted on these polymers and reported in a previous study [41], those results revealed that in the case of AL-OA, the ratio of syringyl to guaiacyl units in lignin (S/G) is 2.73, which is higher compared to the value of 2.11 obtained from AL-EU. This indicates that in oak lignin, there is a greater quantity of syringyl units and, consequently, a higher presence of methoxy groups. Such a condition could promote an increase in the hydrophobicity of lignin, thereby resulting in a greater affinity with the oily medium used in the OCA analysis.

### 3.4. ATR-FTIR Results 

**Figure 9:**
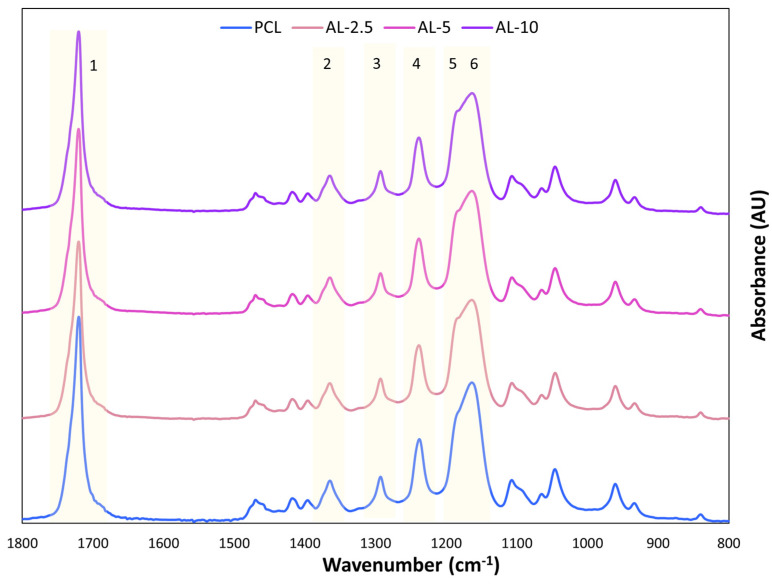
ATR-FTIR spectra comparison of the membrane PCL, AL-2.5, AL-5, and AL-10. PCL-specific peaks assignment: stretching vibration of the –C=O group (band 1), deformation vibrations of the –CH_2_ groups (band 2), stretching vibrations of C–O and C–C groups in the crystalline phase (band 3), asymmetric stretching vibrations of C–O–C (band 4), symmetric stretching vibrations of C–O–C (band 5), and stretching vibrations of C–O and C–C groups in the amorphous phase (band 6).

Spectral profiles were obtained by ATR-FTIR analysis for both lignin grades deposited on PCL. Only the data for AL-EU are plotted in Figure 9, whereas the dataset for AL-OA is omitted because it is practically indistinguishable from the AL-EU. The FTIR spectra of PCL membranes coated with lignin were compared to the spectra of the uncoated PCL membrane. In Figure 9, the distinct stretching and bending signals of PCL are identifiable in all spectra, i.e., within Band 1 (at 1726 cm^−1^) there is a stretching vibration of the –C=O group. Additionally, asymmetric and symmetric stretching vibrations of C–O–C can be observed at 1242 cm^−1^ (band 4) and 1186 cm^−1^ (band 5), stretching vibrations of C–O and C–C groups in the crystalline and amorphous phases at 1295 cm^−1^ (band 3) and 1167 cm^−1^ (band 6), and deformation vibrations of the –CH_2_ groups at 1370 cm^−1^ (band 2) [54]. Instead, no significant signal related to lignin could be observed, with no change in either intensity or area of the absorption peaks at any lignin deposition between 2.5 and 10 min, thus suggesting that the coating is too thin for this analysis.

**Figure 10:**
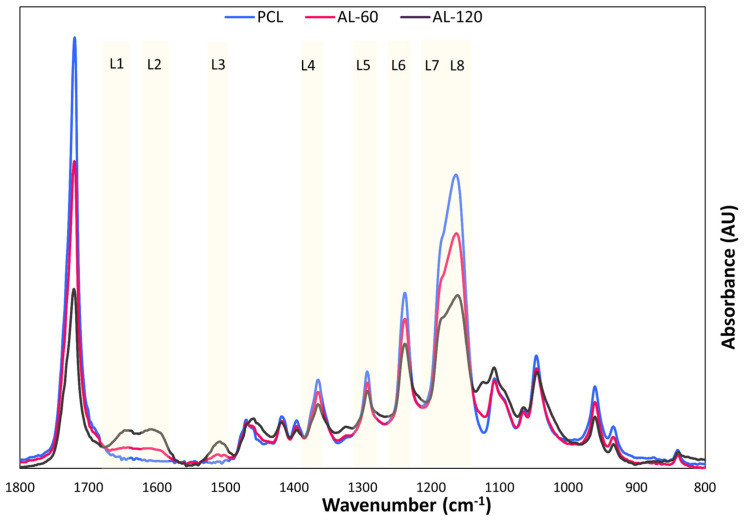
ATR-FTIR spectra comparison of the membrane PCL, AL-60, and AL-120. Lignin-specific peaks assignment: aromatic skeletal vibrations C=O stretching (band L1), C=C aromatic ring vibration (S > G) (band L2), C=C aromatic ring vibration (G > S) (band L3), aliphatic C-H stretching vibrations in CH_3_ (band L4), guaiacyl ring breathing (band L5), C−C, C−O, and C=O stretching in condensed G units (band L6), CH elongation in the G ring (band L7), and the typical S ring CO stretching (band L8).

On the other hand, FTIR analysis of membranes coated with EU-60 and EU-120 in Figure 10 highlighted a clear effect of the lignin. The spectra indeed showed a decrease in the absorption intensity of characteristic PCL peaks, accompanied by the appearance of lignin-specific bands such as L1, L2, and L3. These bands are indicative of aromatic skeletal vibrations: C=O stretching, C=C aromatic ring vibration (S > G), and C=C aromatic ring vibration (G > S). In the case of bands from L4 to L7, a decrease in signal intensity is observed with increasing coating deposition time. This suggests that the extended deposition time of lignin coating may weaken the characteristic PCL signals in this region, favoring instead the specific vibrations of lignin. These deformation bands represent aliphatic C−H stretching vibrations in CH_3_ (L4), guaiacyl ring breathing (L5), C−C, C−O, and C=O stretching in condensed G units (L6), CH elongation in the G ring (L7), and the typical S ring CO stretching (L8) [41].

### 3.5. Antimicrobial Activity

While the main focus of this work is on manufacturing aspects, a biological assessment was designed and performed to assess the antibacterial activity due to the lignin coating, limiting the scope to a representative Gram-positive bacterial strain, i.e., *S. aureus,* and a representative Gram-negative bacterial strain, i.e., *E. coli*. The antibacterial investigation was conducted against Gram-positive and Gram-negative bacteria, although lignins were found to be more effective against Gram-positive bacteria than against Gram-negative bacteria such as *Klebsiella* sp. [55]. 

**Figure 11:**
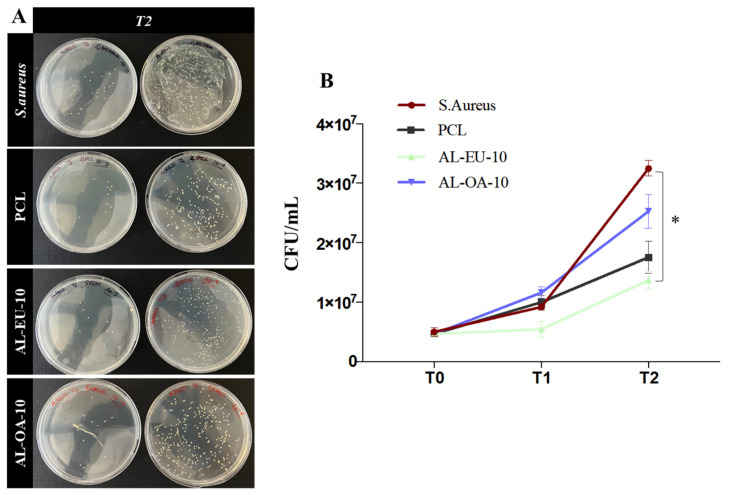
(**A**) Biological experiment 1—Petri dishes obtained from *S. aureus* culture without any mat (control) and cultures with blank PCL and PCL-Lignin mats at two different serial dilutions (10^−5^ and 10^−4^) and last sampled time (3 h); (**B**) Bacterial population (CFU/mL) measured for control, PCL, and coated samples at different time points: 0 (T0), 1.5 (T1), and 3 (T2) hours (* *p* < 0.05).

Our biological study is divided into two parts. At first, we compared three experimental conditions, for three time points (i.e., 0, 1.5, and 3 h), namely: bare PCL, AL-OA-10, and AL-EU-10. The results are shown in Figure 11. Such a biological assay was conducted to gain preliminary insight about the antibacterial performance; this was evaluated against the selected bacterial strain as representative for Gram-positive. As expected, the *S. aureus* culture in the absence of any material (positive control) kept growing steadily up to 3 h, rising from the initial concentration of 5 × 10^6^ CFU/mL up to 3.2 × 10^7^ CFU/mL. Instead, the blank PCL sample induced a weak reduction in bacterial growth. Between the two lignin-coated membranes, only AL-EU-10 showed a sizable antibacterial response, with a concentration of 1.3 × 10^7^ CFU/mL at T2 (Figure 11 and Table 8) and a moderate, yet sustained, antimicrobial action throughout the time interval T1-T2 of 1.5 h. This represents a promising and encouraging result, recalling that the AL-EU-10 sample has a lignin density of just 0.014 mg/cm^2^ (ref. Table 4). 

**Figure 12:**
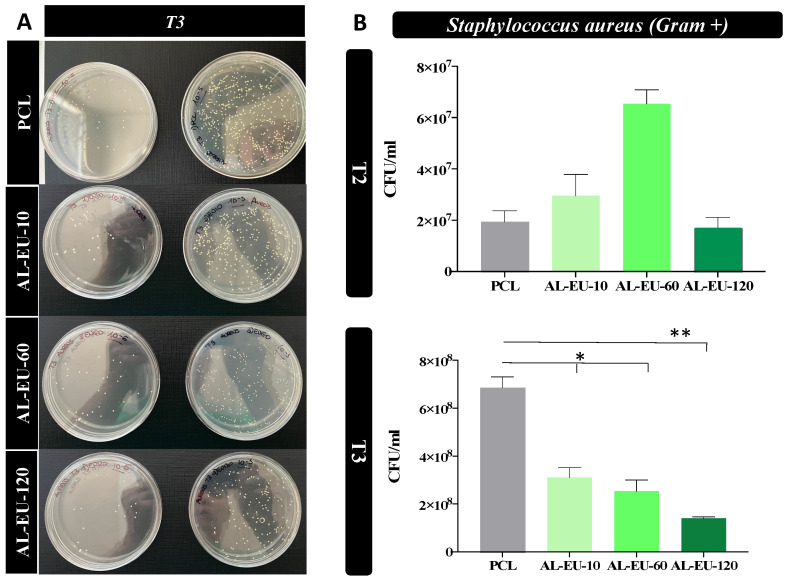
(**A**) Petri dishes obtained from *S. aureus* cultures with blank PCL and PCL-Lignin mats at two different serial dilutions (10^−5^ and 10^−6^) and last sampled time (4.5 h); (**B**) *S. aureus* bacterial population (CFU/mL) measured for PCL and coated samples at different time points: 3 (T2) and 4.5 (T3) hours (* *p* < 0.05 or ** *p* < 0.01).

After determining the precise species of the lignin to be utilized, to expand on this baseline, a second biological experiment against *S. aureus* and *E. coli* was designed to screen out the behavior of AL-EU for longer deposition times, for 10, 60, and 120 min, while monitoring the bacterial growth for 1.5 (T1), 3(T2), and 4.5 (T3) hours. In the experiment against Gram-positive, after 4.5 h, both AL-EU-10 and AL-EU-60 samples showed antimicrobial action by reducing *S. aureus* cellular density by ¼ Log (about 2.1 and 2.7 times, respectively) compared to the bare PCL (ref. Figure 12 and Table 9). The effect on bacterial growth of AL-EU-120 (at 0.165 mg/cm^2^ lignin density) was markedly greater, leading to a steeper decrease in bacterial counts of almost ½ Log, i.e., about 4.8 times compared to bare PCL. The dose-dependent impact of lignin deposition on *S. aureus* is significant.

**Figure 13:**
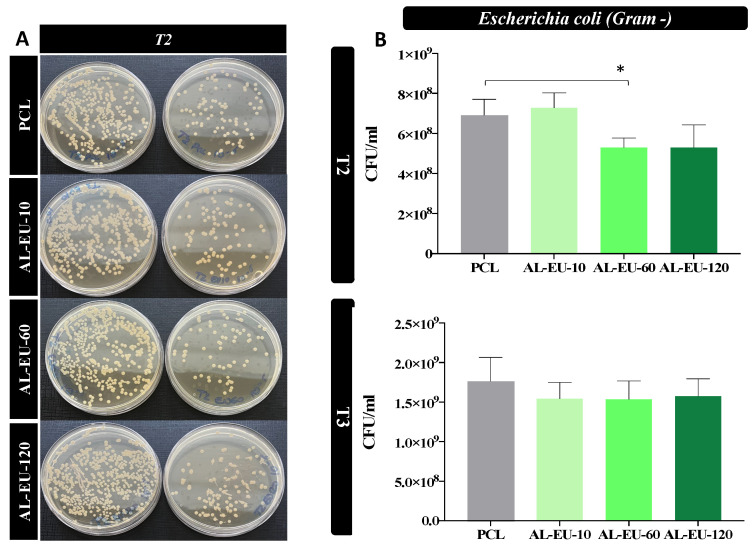
(**A**) Petri dishes obtained from *E. coli* cultures with blank PCL and PCL-Lignin mats at two different serial dilutions (10^−5^ and 10^−6^) and the second last sampled time (3 h); (**B**) *E. coli* bacterial population (CFU/mL) measured for PCL and coated samples at different time points: 3 (T2) and 4.5 (T3) hours (* *p* < 0.05).

In the experiment against Gram-negative, both AL-EU-60 and AL-EU-120 samples exhibited antimicrobial activity after 3 h. However, only AL-EU-60 demonstrated a significant reduction in *E. coli* cellular density, achieving a decrease of 1/6 Log (approximately less than 2 times) compared to the untreated PCL, as indicated in Figure 13 and Table 10. The impact on bacterial growth became only slightly apparent after 4.5 h, with all samples exhibiting a modest, but not significant, reduction in bacterial counts compared to bare PCL. The deposition of lignin against *E. coli* is gradual and extremely subtle.

Despite AL-EU demonstrating its potential as an antibacterial agent against selected strains, it has been observed that its inhibitory effect on microbial growth is more pronounced against *S. aureus* compared to *E. coli*. This result aligns with a study conducted by Gregorova et al. [56], which highlights the antibacterial activity of lignin against both *S. aureus* and *E. coli* through agar diffusion tests. In their case as well, the inhibitory effect is observable on both selected strains, but *E. coli* shows greater resistance to the antibacterial action of lignin. This suggests that this effect may be attributed to the complex structure of the cell walls found in Gram-negative bacteria. Specifically, this resistance could be linked to the presence of the peptidoglycan layer on the outer membrane, which in E. coli is approximately 6–10 times thicker than in *S. aureus*, facilitating the entry of lignin into the bacterial cell [57]. This limited antibacterial effect against Gram-negatives was observed in another previous study where wood samples coated with a lignin-based formulation were subjected to bacterial attack by *Pseudomonas aeruginosa*. In this case, bacterial colonies were numerous and well-developed, indicating that the coating did not demonstrate any antimicrobial effect [58].

In conclusion, the results from these two experiments suggest that, in principle, significant antibacterial activity can be achieved for relatively low lignin densities (here, in the order of ~0.1 mg/cm^2^) and can be increased by increasing the lignin density. In the literature, the antibacterial effects of lignin prepared by different fractionation methods were already investigated, with reports of an antibacterial effect of the lignin obtained by the acidolysis method higher than what we obtained. While more work is needed to perform a direct comparison to make and test such diverse lignin in the context of coating and under the same conditions, we observe that acidolysis leads to a lignin grade with greater exposure of active functional groups, such as phenolic hydroxyl and carboxyl groups [59], compared to the lignin examined in this work.

While the results indicate that lignin could indeed be further developed for antifouling applications and in filtration media, some performance gaps remain. Clearly, the antibacterial performance is considerably lower than other traditional mainstream options, such as, for example, the Ag nanocoating by magnetron sputtering, reported in recent literature by many and also by our group in the context of innovative nanocoatings [15,60]. However, key advantages of the proposed lignin-based antifouling coating strategy vs. Ag nanocoatings would include the following: (i) virtually no limitations in terms of scalability; (ii) semi-continuous manufacturing at ambient pressure in roll-to-roll mode; (iii) replacement of a metal-oxide coating by a biopolymer coating, which is more environmentally friendly and a definite advantage in the pervasive framework of circular economy. Hence, despite the lower performance achieved currently, the possibility of fabricating, on the same single electrospinning platform, an entire layered filter endowed with an antifouling coating from lignin represents an appealing and timely option with great potential impact.

Future work could expand on this investigation along many fronts and, for example, should include a more in-depth antibacterial evaluation to further characterize and optimize this new lignin coating for antifouling applications. Specifically, aside from studying the bacteria *E. coli*, which falls within the research scope, similar to the previously mentioned Ag nanocoating [15,25], one can explore the impact on other representatives within this bacterial group as well as against the Gram-positive group, as has already been carried out for *S. aureus.*

## 4. Conclusions

We investigated and proved the feasibility of a two-step electrospinning fabrication of a biocompatible and biodegradable polymer-on-paper layered system made of electrospun PCL fibers subsequently coated with electrosprayed lignin, all in one production line. The manufacturing of pure lignin coatings by solution electrospraying appears to be novel. In our investigation, lignin was responsible for two main functional properties: (i) the increase in wettability of the substrate towards both polar and apolar liquids, displaying a characteristic amphiphilic effect; (ii) the newly acquired antibacterial properties over the bare PCL membrane. The results achieved with either AL-OA or AL-EU grade, for relatively small lignin densities of order 0.1 mg/cm^2^, indicate that those properties can be tuned with relative ease by the amount of deposited lignin, which makes for an interesting alternative to other surface processing options entailing chemical transformations or physical transformations (e.g., plasma treatments). Also, our data highlight that lignin coatings did not change the overall porosity (a bulk property) of the membrane but reduced surface roughness and moderately reduced air permeability as lignin density increased, which is crucial for filtration applications.

Although the current antibacterial effectiveness of these coated surfaces is significantly inferior to well-established alternatives, such as Ag coatings, there is unquestionably potential for enhancement in these initial endeavors. For instance, when considering the efficacy against Gram-positive and Gram-negative bacteria, i.e., *S. aureus* and *E. coli,* there is certainly room for improvement compared to established options like said Ag coatings. More importantly, the deployment of electrosprayed lignin coating is worth exploring and developing further due to its scalability and potential to be deployed industrially in high-throughput manufacturing of sustainable layered membranes with antibacterial and antifouling properties, not just in the field of filtration. Finally, by highlighting the difference between two acidolysis lignin grades, i.e., *Quercus cerris* L. (AL-OA) and *Eucalyptus camaldulensis* Dehnh (AL-EU) lignins, our study points out that better lignin grades can be in principle carefully selected and engineered upstream to optimize the subsequent electrospraying process. Thus, the capability to make pure lignin coatings by electrospraying opens up and supports the possibility of using and reusing this natural polymer, overabundant and available from wood byproducts, for filtration and for circular economy applications in general. 

## Figures and Tables

**Table 1:** Main process parameters for 2-step electrospinning/electrospraying coating of PCL and AL. Process temperature and RH were kept at 25° C and 40%, respectively, for all steps and samples.

Electrospinning/ Electrospraying	Deposition Time (min)	FR (mL/h)	Distance (mm)	V (KV)	Substrate
*Step 1:* PCL	60	6	150	20	Paper filter
*Step 2:* Lignin	2.5/5/10/60/120	0.75	130	25/−30	PCL

**Table 2:** Treatments from the 2-step electrospinning and corresponding label in this study, AL-EU and AL-OA, correspond to lignin from Eucalypt and Oak, respectively.

Treatment	Label	Definition
1	PCL	basic membrane with PCL coating (step 1 only)
2	AL-EU-2.5	PCL coated for 2.5 min with AL-EU
3	AL-EU-5	PCL coated for 5 min with AL-EU
4	AL-EU-10	PCL coated for 10 min with AL-EU
5	AL-EU-60	PCL coated for 60 min with AL-EU
6	AL-EU-120	PCL coated for 120 min with AL-EU
7	AL-OA-2.5	PCL coated for 2.5 min with AL-OA
8	AL-OA-5	PCL coated for 5 min with AL-OA
9	AL-OA-10	PCL coated for 10 min with AL-OA
10	AL-OA-60	PCL coated for 60 min with AL-OA
11	AL-OA-120	PCL coated for 120 min with AL-OA

**Table 3:** Comparison of electrospun layer thickness (net of paper filter); SD: standard deviation.

Deposition Time of Lignin (min)	Mean Thickness ± SD (μm)	
	Eucalypt	Oak
AL-2.5 (+PCL)	52.95 ± 2.90	58.08 ± 11.70
AL-5 (+PCL)	61.23 ± 5.07	61.85 ± 8.87
AL-10 (+PCL)	49.70 ± 7.04	65.28 ± 2.05
AL-60 (+PCL)	42.75 ± 9.59	38.55 ± 9.23
AL-120 (+PCL)	43.77 ± 8.46	49.00 ± 5.02

**Table 4:** Surface density (per g/cm^2^) and thickness of the lignin coating deposited onto the PCL membranes.

ID Sample	Time Deposition of Lignin (min)	Quantity of Lignin/Area PCL Membrane (mg/cm^2^)	Thickness of Lignin (nm)
AL-2.5	2.5	0.003	24.47
AL-5	5	0.006	48.95
AL-10	10	0.014	97.90
AL-60	60	0.082	587.41
AL-120	120	0.165	1174.81

**Table 5:** Porosity (%) result for each sample.

ID Sample	Porosity ± SD (%)
PCL	72.22 ± 6.98
AL-EU-2.5	67.96 ± 9.40
AL-EU-5	62.55 ± 2.89
AL-EU-10	68.88 ± 3.59
AL-EU-60	74.40 ± 4.78
AL-EU-120	67.09 ± 2.00
AL-OA-2.5	64.42 ± 4.96
AL-OA-5	68.13 ± 6.23
AL-OA-10	72.77 ± 5.90
AL-OA-60	77.27 ± 1.26
AL-OA-120	69.94 ± 6.56

**Table 6:** Air permeability test results by a Gurley densometer.

ID Sample	Permeability ± SD (cm/s)
Filter paper	1.20 ± 0.17
PCL	0.91 ± 0.08
AL-EU-2.5	0.57 ± 0.06
AL-EU-5	0.49 ± 0.03
AL-EU-10	0.68 ± 0.05
AL-EU-60	0.68 ± 0.05
AL-EU-120	0.55 ± 0.01
AL-OA-2.5	0.71 ± 0.05
AL-OA-5	0.64 ± 0.07
AL-OA-10	0.64 ± 0.03
AL-OA-60	0.67 ± 0.02
AL-OA-120	0.58 ± 0.02

**Table 7:** Comparison of the result measurements of water contact angle (WCA) and oil contact angle (OCA).

ID Sample	WCA(SD) (°)	OCA(SD) (°)
PCL	131.93 ± 3.34	19.15 ± 2.68
AL-EU-2.5	94.96 ± 22.95	25.09 ± 1.05
AL-EU-5	116.07 ± 12.76	22.27 ± 2.52
AL-EU-10	116.70 ± 8.13	17.81 ± 1.66
AL-EU-60	*	16.67 ± 3.18
AL-EU-120	*	18.11 ± 2.48
AL-OA-2.5	117.13 ± 7.22	21.28 ± 3.24
AL-OA-5	117.0 3 ± 3.87	17.89 ± 6.99
AL-OA-10	115 ± 6.45	14.56 ± 2.69
AL-OA-60	*	*
AL-OA-120	*	*

*: Measurement cannot be carried out due to complete sample absorption.

**Table 8:** Biological experiment 1—Data are reported as mean values in the table and as mean value ± SEM from two independent experiments, each in triplicate, in the graph.

Time (h)	*S. aureus* CFU/mL (Means)			
	*S. aureus*	PCL	AL-EU-10	AL-OA-10
T0	5 × 10^6^	4.6 × 10^6^	4.6 × 10^6^	4.6 × 10^6^
T1	9.3 × 10^6^	9.1 × 10^6^	5.4 × 10^6^	1.2 × 10^7^
T2	3.2 × 10^7^	1.7 × 10^7^	1.3 × 10^7^	2.5 × 10^7^

**Table 9:** Biological experiment 2 with *S. aureus*. Data are reported as mean values in the table and as mean value ± SEM from two independent experiments, each in triplicate, in the graph.

Time (h)	*S. aureus* CFU/mL (Means)			
	PCL	AL-EU-10	AL-EU-60	AL-EU-120
T0	1.1 × 10^6^	1.1 × 10^6^	1.1 × 10^6^	1.1 × 10^6^
T1: 1.5	5.5 × 10^6^	2.2 × 10^6^	4.6 × 10^6^	8.2 × 10^6^
T2: 3	1.9 × 10^7^	2.9 × 10^7^	6.5 × 10^7^	1.7 × 10^7^
T3: 4.5	6.8 × 10^8^	3.1 × 10^8^	2.5 × 10^8^	1.4 × 10^8^

**Table 10:** Biological experiment 2 with *E. coli*. Data are reported as mean values in table and as mean value ± SEM from two independent experiments, each in triplicate, in the graph.

Time (h)	*E. coli* CFU/mL (Means)			
	PCL	AL-EU-10	AL-EU-60	AL-EU-120
T0	5.2 × 10^6^	5.2 × 10^6^	5.2 × 10^6^	5.2 × 10^6^
T1: 1.5	4.2 × 10^7^	3.2 × 10^7^	2.9 × 10^7^	3.0 × 10^7^
T2: 3	6.9 × 10^8^	7.2 × 10^8^	5.3 × 10^8^	5.3 × 10^8^
T3: 4.5	1.7 × 10^9^	1.5 × 10^9^	1.5 × 10^9^	1.5 × 10^9^

## Data Availability

Data are contained within the article and Supplementary Materials.

## Supplementary Materials

The following supporting information can be downloaded at: https://www.mdpi.com/article/10.3390/polym16050674/s1, Figure S1: Cross section of PCL membrane coated with lignin: AL-EU-2.5 (A), AL-EU-5 (B), AL-EU-10 (C), AL-EU-60 (D), AL-EU-120 (E), AL-OA-2.5 (F), AL-OA-5 (G), AL-OA-10 (H), AL-OA-60 (I), AL-OA-120 (L).
