# Supplementary material for: Electrospun PCL Filtration Membranes Enhanced with an Electrosprayed Lignin Coating to Control Wettability and Anti-Bacterial Properties
Source: Polymers (Basel). 2024 Mar 1;16(5):674. doi: 10.3390/polym16050674 (PMC10934707; doi:10.3390/polym16050674)
Supplement: Supplementary file 1 [file polymers-16-00674-s001.zip › polymers-2864930-supplementary.pdf]

polymers

MDPI

Supplementary material

Electrospun PCL filtration membranes enhanced with an electrosprayed lignin coating to control wettability and anti-bacterial properties

Sara Bergamasco [1], Noemi Fiaschini [2], Luis Alexander Hein [2], Marco Brecciaroli [3], Roberta Vitali [4], Manuela Romagnoli [1,*] and Antonio Rinaldi [5,*]

[1] Department for Innovation in Biological, Agro-Food and Forest Systems (DIBAF), University of Tuscia, Via San Camillo de Lellis snc, 01100 Viterbo, Italy; sara.bergamasco@unitus.it (S.B.); mroma@unitus.it (M.R.)
[2] NANOFABER S.r.l., Via Anguillarese 301, 00123 Rome, Italy; noemi.fiaschini@nanofaber.com (N.F.); luis.hein@nanofaber.com (L.A.H.)
[3] Simitecno srl, Rome, Italy; marco.brecciaroli@simitecno.it (M.B.)
[4] SSPT-TECS-TEB Laboratory, ENEA—Italian National Agency for New Technologies, Energy and Sustainable Economic Development, Via Anguillarese 301, 00123 Rome, Italy; roberta.vitali@enea.it (R.V.)
[5] SSPT-PROMAS-MATPRO Laboratory, ENEA—Italian National Agency for New Technologies, Energy and Sustainable Economic Development, Via Anguillarese 301, 00123 Rome, Italy; antonio.rinaldi@enea.it (A.R.)

* Correspondence: mroma@unitus.it (M.R.); antonio.rinaldi@enea.it (A.R.)

Cross section analysis

The cross-sectional views of the samples were observed using scanning electron microscopy (SEM), wherein the samples were analysed without coating with conductive material and were affixed onto an adhesive support such as carbon tape. The image below (Figure S1) depicts sections of all 10 samples of electrospun PCL membranes coated with electrosprayed eucalyptus (EU) and oak (OA) lignin. The analysis reveals a porous structure of the material, attributed both to the presence of the filter paper (backing material) and the electrospun PCL membrane. The images of the cross-sectional views reveal good adhesion between the materials and no interpenetration phenomena. The layer consisting of lignin nanoparticles cannot be observed; however, it is visible when analysing horizontal sections of the samples (Figure 1 article), as lignin nanoparticles tend to interpenetrate within the mesh structure of PCL.

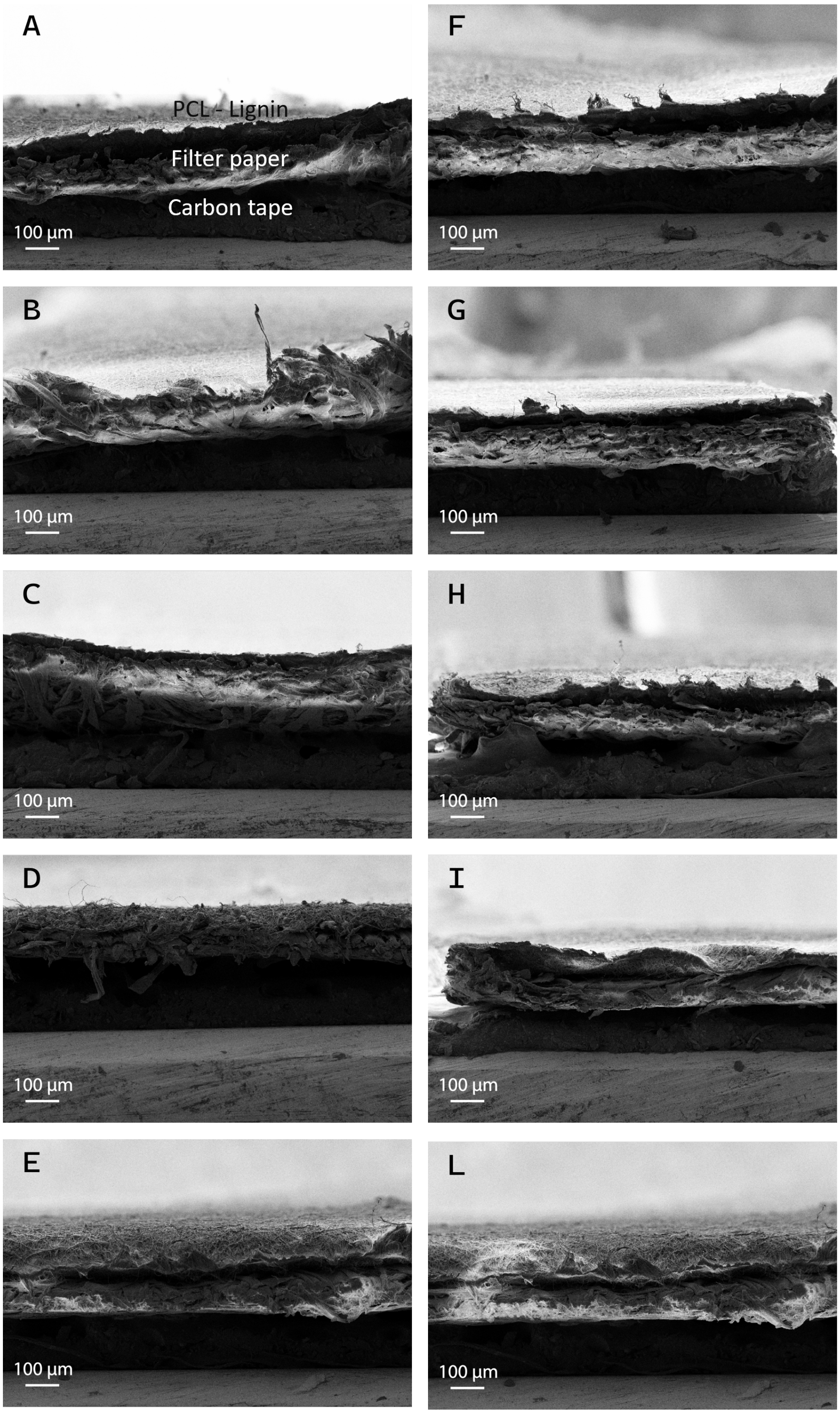

Figure S1. Cross section of PCL membrane coated with lignin: AL-EU-2.5 (A), AL-EU-5 (B), AL-EU-10 (C), AL-EU-60 (D), AL-EU-120 (E), AL-OA-2.5 (F), AL-OA-5 (G), AL-OA-10 (H), AL-OA-60 (I), AL-OA-120 (L).
